# Three-Dimensional-Printed Instrument for Isothermal Nucleic Acid Amplification with Real-Time Colorimetric Imaging

**DOI:** 10.3390/mi15020271

**Published:** 2024-02-14

**Authors:** Tiffany R. Layne, Anchi Scott, Larissa L. Cunha, Rachelle Turiello, James P. Landers

**Affiliations:** 1 Department of Chemistry, University of Virginia, Charlottesville, VA 22904, USA; laynetr@alumni.vcu.edu (T.R.L.); rat3a@virginia.edu (R.T.); jpl5e@virginia.edu (J.P.L.); 2 Department of Mechanical and Aerospace Engineering, University of Virginia, Charlottesville, VA 22903, USA; 3 Department of Pathology, University of Virginia, Charlottesville, VA 22908, USA

**Keywords:** 3D printing, isothermal, loop-mediated isothermal amplification, image analysis, forensic body fluid identification, Raspberry Pi, LabVIEW

## Abstract

Isothermal amplification methods have become popular in research due to the simplicity of the technology needed to run the reactions. Specifically, loop-mediated isothermal amplification (LAMP) has been widely used for various applications since first reported in 2000. LAMP reactions are commonly monitored with the use of colorimetry. Although color changes associated with positive amplification are apparent to the naked eye, this detection method is subjective due to inherent differences in visual perception from person to person. The objectivity of the colorimetric detection method may be improved by programmed image capture over time with simultaneous heating. As such, the development of a novel, one-step, automated, and integrated analysis system capable of performing these tasks in parallel is detailed herein. The device is adaptable to multiple colorimetric dyes, cost-effective, 3D-printed for single-temperature convective heating, and features an easy-to-use LabVIEW software program developed for automated image analysis. The device was optimized and subsequently validated using four messenger-RNA targets and mock forensic samples. The performance of our device was determined to be comparable to that of a conventional thermal cycler and smartphone image analysis, respectively. Moreover, the outlined system is capable of objective colorimetric analysis, with exceptional throughput of up to 96 samples at once.

## 1. Introduction

Loop-mediated isothermal amplification (LAMP) was first reported in 2000 as a novel, highly specific nucleic acid amplification method that is able to generate target sequences at constant temperatures in under an hour [1]. Since then, LAMP has been utilized for various clinical and forensic applications [2,3,4,5]. LAMP involves a set of four to six primers that recognize multiple regions of the target sequence and a Bst polymerase with intrinsic high strand displacement activity [1,6]. As an isothermal amplification method, LAMP assays require a rudimentary heating source only (e.g., water bath, heat block) and eliminate the need for costly thermocycling instrumentation. Much of the original LAMP research [1,2,3,4,5] focused on methods to further increase specificity and reaction speeds, which can be accomplished by including additional primers (i.e., loop primers) that anneal to the loop ends of the main target structure [7]. In addition, various methods for the detection of LAMP amplicons have been demonstrated in the literature [4,8,9]. Visual detection is commonplace and can include observing color change with colorimetric dyes or visual assessment of solution turbidity. Furthermore, quantitative analysis using fluorescence detection and intercalating dyes is often employed [10,11,12,13]. However, employing real-time fluorescence detection is limited by both the hardware (instrumentation) and “chemware” (fluors)—both of which can be costly.

Colorimetric LAMP detection relies on the perception of color change to discriminate between positive and negative reactions. Dyes routinely used for the detection of LAMP products include hydroxynaphthol blue (HNB), phenol red (PR), malachite green, etc. [14], which all have a characteristic chromogenic response to changes in the reaction environment. For example, HNB, a metal-based indicator, changes from “violet” to “sky blue” as free Mg^2+^ binds with pyrophosphates and precipitates out of solution [15]. While colorimetric dyes have been used in a variety of research applications, these endpoint assays can be highly subjective due to variations in user perception of color, which differs significantly from one individual to another [14]. Although endpoint analysis has been used extensively over the last half century, recent research has been trending toward using real-time analysis to obtain a more rapid response and enable quantification of results based on signal intensity. While colorimetric detection is inherently less sensitive than detection via fluorescence, the shift from endpoint to real-time analysis presents an opportunity to minimize the impact of this limitation. As such, the development of a one-step, automated, and integrated system capable of combining the operation and real-time colorimetric detection of LAMP reactions is desired.

Real-time colorimetric analysis can be achieved by capturing reaction images in real time over a predefined period of time. Additionally, image analysis utilizing a variety of color spaces can be facilitated by using a smartphone or small camera that probes the reactions and then exploits software to analyze the color of the reaction in, e.g., the RBG and HSB color spaces [16,17]. It is imperative that the camera, camera settings, and lighting remain consistent over the duration of image capture. Lighting, in particular, must be consistent, as the image “color” detected can be affected by the type of light—white, sunlight, and incandescent, among others—as well as “stray” light from the environment [16]. Concordant conditions can be assured by capturing smartphone images in “manual” mode rather than the default “auto” settings, and software routinely described in the literature for image analysis is Image J [18,19], which features a powerful suite of tools that can be used to analyze an image. For example, the region of interest (ROI) can be selected and then cropped for focused colorimetric analysis (see ref. 16 for details). An image can also be “tinted” to enhance subtle color variations, thereby allowing for improved detection of minute differences in color and increased sensitivity [20,21]. This type of analysis allows for programmed, objective, and consistent measurement of reaction-specific color change over long periods to quantitate reaction progression. In addition to the obvious utility, such a platform technology can help address the inherent deficiencies in the sensitivity of colorimetric analysis as compared with fluorescence methods.

Here, we describe an innovative analytical technology that capitalizes on colorimetric analysis to advance LAMP as an emergent amplification method that could be adapted to a broad range of applications. The system is homebuilt and 3D-printed, with COTS (commercial off-the-shelf) materials that, when assembled, provide a system for isothermal Amplification with Automated Colorimetry—the isoTAAC system—for high throughput analysis. To characterize the isoTAAC system, we utilize a previously described LAMP method for body fluid identification that targets four mRNA markers of great value in forensic analysis [22,23]. Routinely, evidence collected from crime scenes is submitted to presumptive and confirmatory tests that lack specificity and generate high rates of false positive and false negative results [24]. For example, the presumptive test for blood (PTMB test) gives a false positive for plant peroxidases due to the nature of the reaction. To address these limitations, we developed a LAMP method for body fluid identification that removes subjectivity and ambiguity and describes the value of coupling LAMP with image analysis and colorimetry [22]. Accordingly, in this report, we detail the development of the isoTAAC system that accepts standard PCR tubes and, through automated convective heating with precise temperature control and real-time image analysis, streamlines the multiplex colorimetric analysis of 96 reactions under isothermal conditions. Color progression in all sample tubes is monitored simultaneously via a Raspberry Pi camera at predefined time points and is demonstrated with various contrived forensic samples with data comparison to that from conventional methods.

## 2. Materials and Methods

### 2.1. Body Fluid Sample Collection

Venous blood (denoted Vb hereafter), collected via a standard venipuncture routine care technique and treated with 5.4 mg of K2EDTA to prevent coagulation, was obtained from the University of Virginia Medical School (Charlottesville, VA, USA) and stored at 4 °C until use. All other donated deidentified semen (Se), saliva (Sa), vaginal fluid (Vf) swabs, and menstrual blood (Mb) swabs were collected using procedures approved by the University Institutional Review Board (IRB). Freshly donated, deidentified Se and Sa samples were collected in sterile specimen containers, and 50 µL aliquots were prepared and stored at −20 °C until needed. Fresh, deidentified Vf and Mb were collected on sterile cotton swabs, dried overnight, and stored at 4 °C. Mock samples (sample number(s) and composition(s) detailed in Table 1) were prepared on either cotton swabs, cotton cloth, jeans, or filter paper and dried for 24 h before testing. The 4-year-old samples were prepared in 2015 by aliquoting 100 µL of neat blood or semen on filter paper, dried for 24 h, and then stored in the dark at room temperature.

### 2.2. RNA Isolation with DNase Treatment

All samples were lysed and extracted using a previously published protocol [25]. For lysis of Vb, Vf, Mb swabs, Sa, or Se samples, 350 µL of Qiagen RLT buffer was combined with 90 µL RNA-free water, 10 µL Proteinase K (Qiagen, Valencia, CA, USA), and 4.5 µL of β-mercaptoethanol (Sigma Aldrich, St. Louis, MO, USA). Input samples, as appropriate for each known single-source RNA target, consisted of 10 µL of Vb, 2 µL of Se, a whole swab of Vf or Mb, 200 µL of Sa, and ¼ swab, a similarly sized cutting, or 2 mm biopsy punch of prepared mock samples. The samples were incubated at 56 °C for 10 min, and then swab and cloth samples (post-lysis) were put through a centrifugation step to retrieve all lysis liquid. The swab and cloth samples were placed in 0.5 mL tubes that had been punctured in the bottom with a 21-gauge needle. The punctured tubes were then placed in a 1.5 mL microcentrifuge tube and centrifuged at a short spin cycle in a tabletop centrifuge for 1–2 s at maximum speed [26,27]. The remaining fluid from the swab and cloth samples was spun through to the 1.5 mL tube and combined with the original lysed sample. The RNeasy Mini kit (Qiagen, Valencia, CA, USA) with the on-column DNase treatment was used for all purifications of samples according to manufacturer instructions. The DNase mix contained 5 µL DNase I (1 unit/µL; New England, Ipswich, MA, USA) and 75 µL DNase I Reaction buffer (New England Biolabs, Ipswich, MA, USA) for each sample. After completing the purification protocol, all samples were eluted in 50 µL nuclease-free water and stored at −80 °C until needed.

### 2.3. Primer Information for Messenger RNA Targets

Human Β-globin (HBB; accession no. NM000518.5) was targeted for Vb identification, cytochrome P450 family 2 subfamily B member 7 (CYP2B7P; accession no. NR_001278.1) for Vf, human semenogelin-1-precursor (SEMG1; accession no. NM003007.4) was used as a Se marker, histatin-3 precursor (HTN3; accession no. NM000200.2) for Sa, and matrix metallopeptidase 10 (MMP10; accession no. NM_002425.2) for Mb detection. The LAMP primer sets were designed using PrimerExplorer V5 and purchased from Eurofins Genomics LLC (Louisville, KY, USA).

### 2.4. Colorimetric LAMP 

The WarmStart^®^ Colorimetric LAMP 2X Master Mix (DNA & RNA) (M1800) (New England Biolabs, Ipswich, MA, USA) was used at half-reaction volume with a 10% input sample volume for all the experiments according to the manufacturer’s instructions. LAMP reactions were heated in the isoThermal Amplification with Automated Colorimetry (isoTAAC) system at 63 and 65 °C for up to 60 min, with primer concentrations of 0.2 µM for F3 and B3, 0.8 µM for LF and LB, and 1.6 µM for FIP and BIP. To determine whether the LAMP reactions were positive or negative for the presence of the intended RNA marker, the first 3–4 min were analyzed and averaged for hue value(s), with 3 standard deviations added to establish a threshold. Samples that resulted in false positive results (i.e., Vb mock sample) were analyzed on Agilent 2100 instrumentation using DNA 1000 series II kits (Agilent Technologies, Santa Clara, CA, USA) to confirm target amplification. A set of Se mock LAMP reactions were further analyzed using an ABAcard^®^ p30 test (Abacus Diagnostics, West Hills, CA, USA) following the manufacturer’s protocol.

### 2.5. isoTAAC System

The isoTAAC system consists of a 6.75 × 7.5 × 7.5 in. 3D-printed acrylonitrile butadiene styrene (ABS) enclosure (i.e., the heating chamber) printed on a Dremel Digilab 3D35 printer (Dremel, Mt. Prospect, IL, USA), a forced convection heating system, an imaging system, direct front lighting, diffused backlighting, an 8.5 × 8.5 × 2.5 in. black polylactic acid (PLA) housing for integrated electronics, and a user interface with an automated image analysis program controlled by a laptop computer. Three strips of white light-emitting diodes (LEDs) are located inside the top of the device and are covered by a diffusive layer. A Raspberry Pi (RPi) camera v2.1 and Raspberry Pi 2 Model B+ v1.2 controller (Raspberry Pi, Cambridge, UK) were used for image capture. A 120C AC Single Phase Heater was attached to the back of the heating chamber in a white ABS holder. Temperature was monitored with a T-type thermocouple connected to the ABS enclosure by a thin tube and placed directly below the 96-well metal plate. Inside the heating chamber are four 25 × 25 × 10 mm 12 V DC fans and two LED strips on either side of the RPi camera. To run LAMP reactions in the isoTAAC system, the user inputs the appropriate parameters (e.g., temperature(s), time(s), etc.) into the software, adds the samples to the instrument, and hits “start” on the software. Real-time data are produced over the course of the 60-min run, with automatic image capture every 60 s. Once the reaction is completed, files are produced at the end in a single run folder and images are analyzed for hue using the exported.csv files.

### 2.6. Mock Study

For the mock case study, approximately 1/4th of the swab, or similarly sized cuttings from samples deposited on fabric, were harvested, sealed in a tube, and assigned a sample ID before being transferred for processing and analysis. Samples were shared for processing and analysis in a blind manner; that is, samples were deidentified for their specified body fluid(s) to prevent bias. For amplification by LAMP on the isoTAAC system, samples were run each at 63 °C and 65 °C given that these were the temperatures previously determined to work best with our panel of LAMP primers for body fluid identification [25].

## 3. Results and Discussion

Interest in LAMP as an isothermal assay continues to increase, and this is mirrored by the increased availability of optimized commercial chemistry kits (NEB, ThermoFisher Scientific, Biosearch Technologies, etc.). The development of several prototype instruments for the detection of LAMP products has been described in the literature [28,29,30]. For example, Papadakis et al. describe a simple but elegant system for the analysis of eight samples with read-out on a tablet, demonstrating its utility for the detection of SARS-CoV-2 [29]. Given the speed, specificity, and colorimetric capability of LAMP assays, a system capable of higher throughput was needed.

### 3.1. Colorimetric Analysis

**Figure 1:**
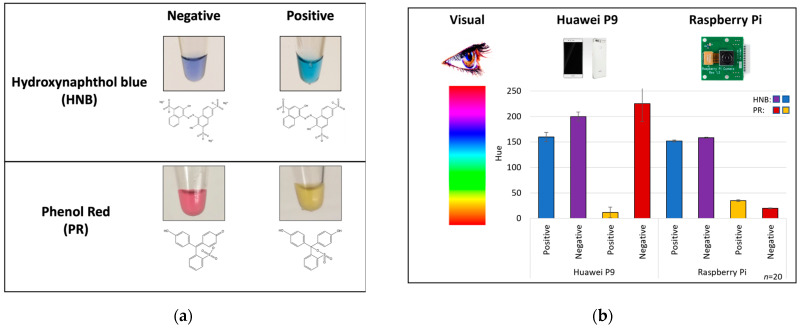
Colorimetric dyes used for the analysis of LAMP reactions. (**a**) HNB changes from purple to blue as the Mg^2+^ concentration decreases in the reaction. PR changes from red/pink to yellow as the pH changes from ~8.4 to ~6.4 in the reaction. (**b**) Color is analyzed differently between the human eye, Huawei smartphone (Huawei, Shenzhen, China), and Raspberry Pi camera. Previously, the Huawei smartphone was used for color analysis, but the integrated system was built with a cost-effective Raspberry Pi camera. The Raspberry Pi camera provides stricter bounds for positive and negative colors than the Huawei smartphone. Each bar represents an average and 3× standard deviation across 20 reactions.

We previously designed a LAMP assay that was optimized for the detection of messenger RNA (mRNA) specific to various body fluids of forensic relevance [22]. In that study, the isothermal assay was facilitated by heating in a conventional thermal cycler, and images were captured using a stand-alone 3D-printed “imaging box” and smartphone (Figure S1). The LAMP reactions were analyzed colorimetrically to determine the presence or absence of a target with either HNB or PR dyes (Figure 1a). HNB changes color from purple (negative) to blue as pyrophosphates, created during base incorporation, react with Mg^2+^ to form a precipitate that is indicative of amplification. Similarly, PR changes color from red/pink (negative) to yellow as the amplification evolves due to the production of H+, which changes the solution pH from ~8.4 to ~6.4. While a number of “color spaces” are available for executing colorimetry [16], hue in the HSB (hue, saturation, brightness) color space was determined to provide the best discrimination for monitoring the progression of amplification (Figure 1b). The hue value reflects a “shade” of a color, with values that span from 0 to 255 where, e.g., PR amplification progression changes the color over a large part of the hue spectrum, from 220 to 40. HNB, on the other hand, changes over a narrow portion, from 200 to 160 during amplification. A threshold was set for both dyes to distinguish negative results (no amplification) from positive results (target-based amplification) by averaging the baseline hue value (first 2 min of the LAMP reaction) and adding three or five standard deviations, depending on which colorimetric dye was used.

**Figure 2:**
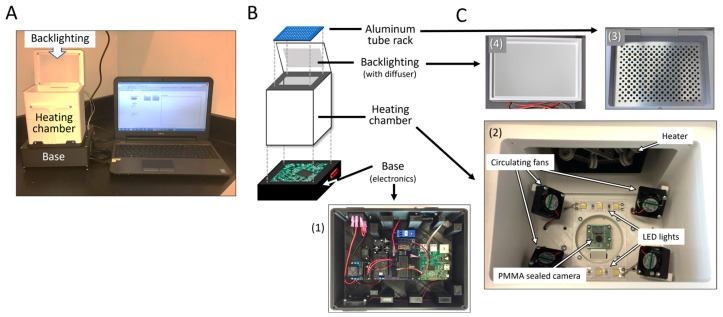
Integrated isoTAAC system for real-time amplification and image analysis. (**A**) The 3D-printed system is connected to a laptop with LabVIEW software version 17.0.1fl via an ethernet cord. (**B**) The system consists of a base, a heating chamber, and a chamber lid with diffused backlighting (**C4**). Below the heating chamber are (**C1**) the electronics necessary for heating and image capture. The heating chamber contains a heating element, 4 fans, and direct front lighting on either side of a Raspberry Pi camera (**C2**). LAMP reactions in 0.1 mL microcentrifuge tubes are seated in a CNC-machined metal plate, which sits on top of the heating chamber (**C3**).

An early version of a “light box” that allowed for the evaluation of static lighting, specifically yellow light vs. white light, was more conducive to colorimetric analysis via ImageJ (Figure S1), and this defined white light as more effective [22]. However, this crude system suffered several disadvantages. One drawback involved the manual transfer of the tubes from the thermal cycler to the “light box”, where image capture and analysis provided a “hue data point” representative of amplification progression for each tube. Even with expedited transfer to and from the image box, fluctuations in sample temperature would result, inevitably affecting the efficiency of the amplification, and potentially enhancing non-specific amplification (NSA). This was highly problematic because the color change from true amplification cannot be distinguished from NSA, which generates a false positive. Another disadvantage of this system was that, for initial purposes, the reaction tubes had to be laid horizontally for image capture and, if this was not performed carefully, the reaction mixture would shift from the apex of the tube to the cap, prohibiting image analysis. If this occurred, the tubes required brief centrifugation, which added to the manual intervention, further exacerbating sample temperature fluctuation and potentially enhancing NSA. To alleviate these shortcomings, a system was built whereby LAMP could be seamlessly combined with detection (isoTAAC) (Figure 2) for one-step isothermal LAMP with real-time colorimetric image analysis.

### 3.2. The IsoTAAC System

#### 3.2.1. Hardware

The isoTAAC system consists of a 3D-printed acrylonitrile butadiene styrene (ABS) enclosure, armed with convection heating, an imaging system, direct front lighting, diffused backlighting, integrated electronics, and a user interface with an automated image analysis program. The accompanying software will be discussed in a later section. Figure 2 provides an overview of the isoTAAC system composed of a base, a heating chamber, a tube holder, and a chamber lid. Figure 2A shows the isoTAAC instrument and, once powered up, the isoTAAC system can be activated to run through a series of “modes” driven by a laptop with LabView software version 17.0.1fl. Figure 2B shows an exploded view of the isoTAAC system. The “base” (Figure 2(C1)) contains all of the electronics required to control the heating, lighting, and image analysis functions via the laptop connected through an ethernet cord. The “heating chamber” has two functional components—one for heating and maintaining the isothermal temperature needed for amplification, and the other is an image capture subsystem. Heating is accomplished with an inexpensive coil-based heater fixed to the outside and positioned at the back of the heating chamber (Figure 2(C2)), and the chamber temperature is recorded with a T-type thermocouple affixed to the chamber wall directly below the metal plate nearest to the reaction tubes. With a chamber volume of 964.40 cm^3^, four small fans positioned at the bottom corners of the chamber were found adequate to assure forced air convection that provides homogenous heating of sample tubes. On the floor of the chamber resides the electronics for image capture and analysis, which include a Raspberry Pi 2 (Model B+ v1.2) camera and printed circuit board mounted in a center recess, with polyethylene terephthalate (PET) covering the electronics. LED strips are located on either side of the camera for bottom-up lighting. The top of the chamber is capped by an opening with a slight recess that accepts a CNC-milled aluminum plate with conical-shaped sample ports for 96 (0.1 mL) microcentrifuge tubes for LAMP amplification, as well as cylindrical heat ports as air vents for heat flow above and below the tubes (Figure 2(C3)). The bottom of the metal plate is painted matte white to alleviate reflection on the metal surface from the LED lights next to the RPi camera during image capture. The metal plate has etchings to denote row letters and column numbers for correct placement. Finally, the lid (Figure 2(C4)) serves to contain heat in the chambers (upper and lower) to minimize fluctuations in the temperature needed for isothermal amplification. Importantly, it contains an embedded bank of LED lights that illuminate the tubes (from the top) through a diffuser membrane.

#### 3.2.2. Isothermal Heating

**Figure 3:**
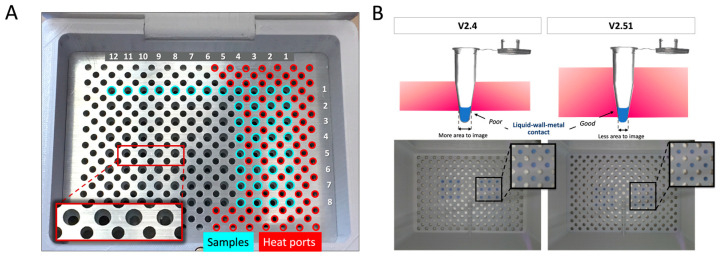
Comparison of different structured metal plates. (**A**) The 6.5 mm metal plate was initially tested with contact ending at a higher part of the tube and then compared to a 9.75 mm metal plate with more contact but less area to image. (**B**) Exemplary images acquired from the camera during heating.

In order for LAMP to be efficient, the reaction tubes must be at the same constant temperature for the duration of the amplification while being visually available for image analysis. This is accomplished in two ways: (1) by providing an environment where the air temperature above and below the sample tubes is equilibrated to the desired temperature, and (2) by using the sample holder to ensure homogenous and constant heating of the reaction mix in the tubes. Figure 3A shows an image of the aluminum plate with the right side highlighting larger sample ports (blue) surrounded in a repeating pattern by smaller heat ports (red). The 12 × 8 array of sample ports offers the capacity for the simultaneous amplification of 96 samples. The abundance of heat ports was designed to enhance heat transfer to the upper chamber(s) to rapidly achieve system equilibration at the isothermal temperature that yields an in-tube temperature of ~68 °C. We evaluated the relationship between the chamber temperature and that in-tube. It became clear that an optimized sample port “draft angle” could enhance intimate contact between the metal and tube and, thus, help maintain a constant in-tube temperature despite slight fluctuation in chamber temperatures. The two metal plates, ~6.5 mm (V2.4) and ~9.75 mm (V2.51) in thickness, were machined to a conical shape to accommodate 0.1 mL microcentrifuge tubes (Figure 3B). With V2.4, the tube projected further from the bottom of the 6.5 mm plate to provide a larger area for effective image analysis, but left most of the sample/wall in contact with heated air, and did not leverage the aluminum plate as a contributor to the in-tube liquid temperature. Hence, with V2.51, the diameter of the drilled port was decreased in the 9.5 mm plate to maximize heat transfer, at the cost of reduced area for imaging. With this design, when the plate was thermostated to a temperature that yielded an in-tube temperature of ~68 °C, a dual heating approach resulted, where the reaction mixture temperature was less affected by slight fluctuations in chamber temperature, owing to the large thermal mass of the aluminum plate. This results in temperature changes over the course of the amplification at a much slower rate.

**Figure 4:**
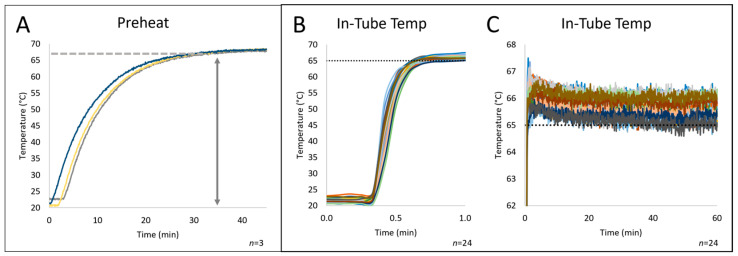
Temperature stability inside the heating chamber of the isoTAAC system. (**A**) Metal block temperature during the 45 min pre-heat step, which achieves the desired temperature. (**B**) The in-tube temperature after being placed in the isoTAAC system. All samples ramp to 65 °C within 30 s and are stable at 65 °C over time. Various wells were monitored to assess the correct temperature.

To test the effectiveness of the V2.51 design, the system was put through the “pre-heat” process without samples to determine the time needed to equilibrate the plate at the isothermal LAMP temperature. With a thermocouple bonded to the top of the plate, Figure 4A shows the kinetics of heating the system. The rate at which the aluminum reaches the desired temperature of ~68 °C is rapid in the initial stages, reaching 65 °C in ~20 min, followed by a slow gradual rise to a temperature of 68 °C over roughly the same timeframe. This stipulated that, prior to LAMP, the asymptotic heating profile for the system necessitated a 45 min “pre-heat”. Once the desired temperature is reached, the software uses a simple feedback loop to facilitate the heating of the chamber to yield an in-tube reaction mixture temperature of 68 °C. Following the “pre-heat” step, Figure 4B shows that placing a sample tube (containing liquid and a thermocouple) into the plate leads to a rapid increase in liquid temperature, reaching the desired temperature within 30 sec—an overlay of 24 runs indicates good reproducibility. Since LAMP is isothermal, maintaining the desired in-tube temperature with minimal variation is important, and Figure 4C shows that achieving the desired T +/− 1 °C was possible.

#### 3.2.3. System Software Control

**Figure 5:**
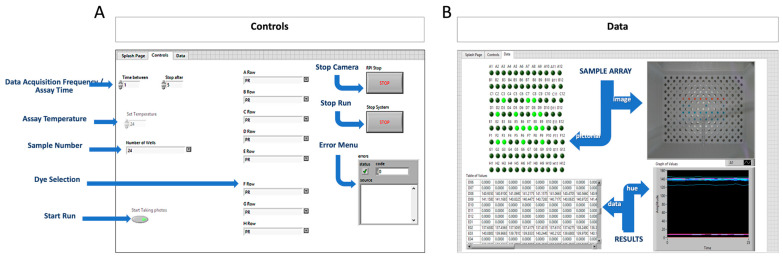
LabVIEW software for image capture and analysis in the isoTAAC system. (**A**) In the Control tab, experimental variables pertaining to overall time, image acquisition time, heat temperature, number of reactions, and colorimetric dye are entered and saved before starting the experiment. (**B**) In the Data tab, the color analysis of all 96 wells is shown in real time. The image will change after a new capture and ~45 s after, the values are shown in table and graphical format. A threshold is calculated by averaging the hue from minutes 0–2 for each reaction and adding 3 or 5 standard deviations. Once a sample has passed the threshold, the location will light up in the sample array.

With the heating capabilities established, in-house code for process control heating synchronized with colorimetric analysis was defined with a combination of Propeller and LabView software. Upon switching “on” the isoTAAC system, the Raspberry Pi controller (programmed with Propeller code) activates the heater and monitors the heating chamber temperature via a Type-T thermocouple to heat to 80 °C; this yields an in-tube temperature of ~68 °C inside 45 min. When LabVIEW is opened, three tabs appear—“Splash Page”, “Controls” and “Data”—where the “Controls” tab allows the user to define the parameters for that run. Figure 5A provides a representation of the laptop screen with “Controls” opened; it is here that the user defines the parameters for “total assay time”, “image acquisition frequency”, “assay temperature”, “number of reactions”, and “type of colorimetric dye”. Additional controls are seen on the right for alerting the user to any errors detected during the run, as well as options to “stop the camera” or “stop the run”. Once the parameters are defined, the user can “start the run”, and data will then be displayed in the “Data” table. The “Data” tab (Figure 5B) provides real-time information on both the sample array and the results. An image of the array is provided directly from the RPi camera, along with a pictorial representation of samples within the array. Colorimetric (hue) data for each tube is graphed in real time, and a table populates with new values every 60 s. With this being a qualitative LAMP result, a “threshold value” is internally calculated from the average hue values over the first 2 min for each reaction, plus three or five standard deviations, depending on which dye is used—this is detailed in Figure S2. If the hue value for a particular sample exceeds the threshold, this is automatically revealed in the pictorial rendering of the array, where the sample location turns a bright green, thus, alerting the user to samples in the 96-tube array that are true “positives” over the course of amplification. In terms of data storage and acquisition, the LabVIEW software creates a text file (.txt) upon run initiation that contains all data from the most recent run and associates it with all the parameters defined on the “Control” table. After each image is produced, it is saved as “Image_X.jpg” for each subsequent 60 s interval (new images are saved as “Image_1.jpg”, “Image_2.jpg”, etc.). The raw hue values for each image are saved in a “comma-separated values” (.csv) file, and the averaged hue values for each image are saved in a separate .csv file. Both of these files contain hue values for all 96 wells over the defined total assay time. The LabVIEW software for the isoTAAC system stores all the relevant data from a run in a single folder that is titled by the user—the user is able to manipulate the raw or averaged data as needed.

#### 3.2.4. Optimizing the Heating Rate and Stabilization

**Figure 6:**
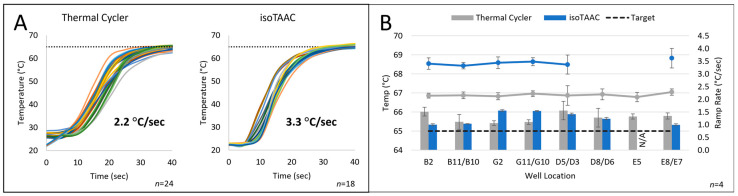
Comparison of integrated isoTAAC system temperatures and ramp rates with a conventional thermal cycler. (**A**) All wells increased to the desired temperature within 30 s on both systems. (**B**) Various wells were analyzed to compare temperature across the 96-well plate in a conventional thermal cycler and isoTAAC system. Each bar is an average of the temperature across 45 min for four days. The ramp rate was higher for the isoTAAC system than the thermal cycler.

After constructing the isoTAAC prototype and associated software, testing was performed to determine the necessary heating chamber temperature, optimize the rapid temperature increase required for the LAMP reactions, and evaluate the temperature stability over relevant time periods. The air temperature in the heating chamber during preheating and amplification was controlled by a feedback loop from a thermocouple mounted to the wall with a small hollow tube just below the metal plate, while the in-tube temperature was monitored using a homebuilt eight-thermocouple system that allowed for simultaneous monitoring of temperature at different locations in the array. For each well, 12.5 µL of water was added and a thermocouple was fed into the water through a hole in a cap sealing each well being tested. Figure 6A shows the curves that represent the liquid heating rate in-tube from eight tubes in the array post-preheating—ramping from room temperature to ~ 65 °C over a 45-min time frame. The isoTAAC system compared favorably to a commercial thermal cycler and, in fact, performed better with an average ramp rate of 3.3 °C/s (versus 2.2 °C/s) and with tighter consistency in the ramp rate (less variation) than the thermal cycler.

In the initial experiments, the set point temperature for the heating chamber was set to the desired isothermal temperature needed for LAMP (e.g., 65 °C), and after a 45 min pre-heat, sample tubes with the reaction mixture were placed into the isoTAAC system array to initiate amplification. However, it was noted that the reactions did not always reach ≥65 °C as rapidly as expected (Figure 6A)—sometimes requiring 10 min or more. Hence, the pre-heat temperature was set 2–3°C higher (e.g., 68 °C) than the desired isothermal temperature (e.g., 65 °C) to offset any heat loss in the system. With this as a standard part of the operating procedure, the in-tube reaction mixture reached the amplification temperature within 30 s. Figure 6B shows both the ramp rate (right axis) and in-tube temperature across eight different locations in the array as determined empirically using an eight-thermocouple system. A baseline in-tube amplification temperature was set to hit 65–66.5 °C for the isoTAAC system, and as shown in the figure, this was maintained consistently by the conventional thermal cycler in most wells for over 45 min (Figure 6A). To reach a comparable in-tube amplification temperature in the isoTAAC system, a chamber temperature of 77 °C was found to be optimal to consistently reach ~ 65–66.2 °C across all wells. This provided ample evidence that, regardless of the location in the 96-tube array, there was consistency in the attained temperature and the rate at which it was reached. Overall, the isoTAAC system was shown to be capable of rapidly heating the LAMP reactions to the desired amplification temperature and, subsequently, maintaining that temperature for the entire 60-min testing period.

We were initially puzzled by the fact that the number of sample tubes analyzed seemed to result in slightly different in-tube temperatures. Knowing that the metal plate contained 209 heat ports and 96 sample ports, it became clear that analyzing a smaller number of sample tubes (e.g., 24) would leave 72 sample ports open and that these would work in concert with the 209 heat ports to enhance the equilibration between the upper and lower chambers. In contrast, a larger number of samples, e.g., 88, would only leave eight sample ports open to augment the temperature equilibration. The data in Figure S3 support the operating procedure strategy that, with the array completely filled (96 tubes), a minor adjustment to the heating chamber set temperature was required, but for fewer than 72 wells, no augmentation was required. We concluded that the simpler, fail-safe approach was to fill all sample ports, regardless of the number of bona fide samples to be analyzed.

### 3.3. Colorimetric Image Analysis

Having demonstrated the ability to provide appropriate thermal conditions for LAMP, attention was directed toward the image capture and analysis portion of the system. Recall that the isoTAAC system was designed to complete LAMP while also providing real-time monitoring to enable non-biased and automated colorimetric image analysis. Early efforts focused on trialing two different cameras for image capture. Interestingly, the representative data demonstrated differences in hue values as a result of camera selection. For example, a comparison of the specifications of the Huawei P9 smartphone camera with the RPi camera in the isoTAAC system is given in Table S1, whereby minor differences are detailed. Given that the biggest change between the two image capture systems involves the camera’s respective focal lengths, we suspect that differences in hue result from color separation related to lateral chromatic aberration [31]. Nevertheless, the P9 smartphone and the isoTAAC system provide comparable results to those obtained from our previous studies with LAMP reactions [22,32].

**Figure 7:**
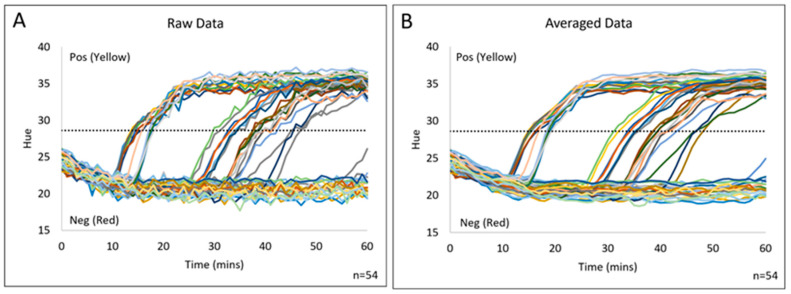
Hue smoothing of phenol red via moving average calculation in Microsoft Excel software. (**A**) The raw image analysis shows how the phenol red hue can vary from minute to minute. (**B**) The averaged image analysis data smooths the transition between time points by averaging the previous two data points and the current data point. Each line represents image analysis from one sample.

Preliminary testing of the complete isoTAAC system with the RPi camera probed 54 body fluid samples for the presence of the previously described mRNA panel [25]. Figure 7A demonstrates the raw output from the real-time monitoring of hue values, which is similar to cycle numbers in real-time PCR. The raw data are further processed in Microsoft Excel Microsoft 365 version using an algorithm that imparts a three-point moving average, providing a “smoothed” output, as seen in Figure 7B. Here, the “threshold hue value” is determined at the 4 min point, allowing the sample to be classified as either “positive” or “negative”. The classification is further based upon a threshold calculation that is described in Figure S2. Following real-time monitoring of the system, both the raw and averaged hue values can be visualized in the “Data” tab of the software and saved in separate .csv files by the user.

**Figure 8:**
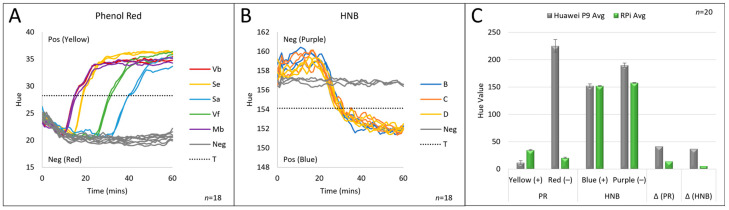
Integrated LAMP runs. (**A**) LAMP with phenol red where the hue values increase if samples become positive. (**B**) LAMP with HNB where hue values decrease if samples become positive. (**C**) The averaged hue values for positive and negative reactions for both indicators and the differential in hue values for each setup.

As a result of the flexible image analysis method, the isoTAAC system can be used with any colorimetric dye selected by the user. Figure 8 shows the data obtained for LAMP reactions using the PR (A) and HNB (B) dyes, where the threshold value was calculated above (PR) or below (HNB) the initial hue values for the dye of choice. These dyes show the bandwidth of capability for this colorimetric image analysis system, owing to the opposite nature of the directionality of the hue value change—low-to-high for PR and high-to-low for HNB. As a result, any colorimetric dye change in a LAMP reaction can be analyzed using the isoTAAC system. The user simply selects the colorimetric dye of choice prior to initiating the LAMP assay, and the software automatically directs the “adding” or “subtracting” of the threshold from the average starting values, as appropriate. A comparison of the isoTAAC image analysis methods with the previously used Huawei P9 camera indicates that the change in PR hue value from negative to positive is more prominent when using the P9 device (Figure 8C). Although the change in hue value is more significant for both dyes using the P9 smartphone, the isoTAAC system was still able to show the color change numerically for both dyes with much smaller standard deviations. This feature indicates that the isoTAAC system performs comparably, or potentially better than the previously designed image analysis method for these dyes [22].

### 3.4. Mock Study Analysis

While colorimetric LAMP is ideal for multiple use cases at the point of need (PON), it is particularly well suited for the identification of deposited body fluids related to forensic casework. In these instances, serological testing is often used to probe for suspected human-derived materials to provide context, support witness testimony, or indicate the presence of DNA for human identification by downstream short tandem repeat (STR) analysis [33]. In these cases, body fluids may be present in isolated circumstances or mixed, requiring testing to deconvolute their signatures, either by immunological assays, colorimetric tests that probe for certain proteins, or nucleic acid tests [34]. In recent years, several analytical tests have been developed that probe for the presence of certain mRNA targets specific to forensically relevant body fluids [35]. As a proof-of-concept, we used the isoTAAC system to probe for these transcriptomic signatures in body fluids including blood, semen, saliva, menstrual blood, and vaginal fluid. Samples spiked with body fluids known to result in false positive results were also included, such as breastmilk and nasal mucosa. A total of twelve mock forensic samples were prepared to approximate those that might be encountered by case working laboratories; while some samples were probed from recently prepared swabs, others were dried and aged for up to four years, or mixed with multiple contributing fluids, often at low input levels. Following the isolation of mRNA from each sample, extracted eluates were probed for the detection of each body fluid in the isoTAAC system using the previously published mRNA targets [25].

Table 1 details the body fluid compositions of each mock sample, numbers 1–12, as well as the body fluids that were experimentally probed for and their expected versus actual results (e.g., positive (+) vs. negative (−)).

A total of 6 of the 12 samples were correctly identified by the isoTAAC system, with the remaining samples producing either false positive or false negative identifications. Despite the seemingly high miss rate, it is important to contextualize these results within the framework of the known transcriptomic idiosyncrasies related to stochastics and specificity. For example, all samples containing semen markers (S1, S2, S7, and S9) were spiked with either a very low concentration of semen or semen aged up to four years; in each of these cases, the RNA input is ostensibly low, which can lead to false negative results [36]. With regard to specificity, many of the body fluids tested show false positive indications; for instance, mock study samples S1, S2, and S12 show false positive indications for Sa and Vf, while the nasal swab spiked with saliva (S4) is mistakenly identified as having a blood marker. In these instances, cross-reactivity is well documented in the literature for transcriptomic targets, whereby nasal, vaginal, and salivary secretions are often misidentified [37]. Markers for the mRNA-based identification of blood are also known to show cross-reactivity with tissues including menstrual blood and saliva [36]. Broadly speaking, transcriptomic analysis for this purpose requires careful optimization of the RNA input, as levels outside of the analytical range can induce false positives and false negatives and enhance the potential for cross-reactivity [36]. For these reasons, it is advisable to target multiple markers for each body fluid; of course, the high throughput possible with the isoTAAC system makes this possible. In retrospect, early testing of the isoTAAC system for mRNA-based body fluid detection with only one target per body fluid was an ambitious first application.

## 4. Conclusions

Overall, the data demonstrate the functionality of a simple, 3D-printed isoTAAC system for LAMP reactions with image analysis. This system provides objective results based on hue color change, similar to quantitative fluorescence detection systems typically used for polymerase chain reactions (PCRs). The hardware and software are user-friendly and compatible with multiple colorimetric dyes. A threshold is calculated with every LAMP run on the isoTAAC system and displayed on the “Data” tab in the software. The isoTAAC system can rapidly heat LAMP reactions from room temperature to the desired amplification temperature and steadily maintain this for more than 60 min. Compared with a conventional thermal cycler, the isoTAAC system demonstrated similar temperature outputs and ramp times across multiple well locations. The isoTAAC system was also able to amplify single-source known samples and mock forensic samples for four mRNA targets for blood, semen, saliva, and vaginal fluid with moderate success.

While the isoTAAC system is a novel first step toward automating body fluid profiling, further improvements are likely required. In the next phase of development, the RPi camera will be replaced with a camera on rails to mimic the camera inside a conventional scanner. This redesign will permit a drastic reduction in the requisite height of the heating chamber, thereby allowing for more rapid preheating and a reduction in the overall time required for each assay. Additionally, the laptop will be replaced with a touchscreen integrated into the top of the isoTAAC system, decreasing its overall footprint. With these changes, the isoTAAC system will be more portable for on-scene or point-of-care analysis with a more rapid overall sample-to-answer time. The isoTAAC system outlined in this paper detailed a rapid, portable, automated objective analysis method for the colorimetric detection of various mRNA targets using a LAMP reaction with the potential for further applicability to a wide range of targets.

## Figures and Tables

**Table 1:** Mock sample identification using the isoTAAC system with RPi image analysis. The red + or − signs denote inconsistent identifications between the expected (E) and actual (A) results.

Sample Number	Mock Sample	Vb		Se		Sa		Vf	
		E	A	E	A	E	A	E	A
1	VF swab spiked with semen (100 µL 1:100)	−	−	+	−	−	+	+	+
2	MB swab spiked with semen (50 µL 1:100)	+	+	+	−	−	+	+	+
3	MB swab	+	+	−	−	−	−	+	+
4	Nasal swab spiked with saliva (100 µL)	−	+	−	−	+	+	−	−
5	VF smear on cloth, spiked with male saliva (100 µL)	−	−	−	−	+	+	+	+
6	Blood smear on jeans	+	+	−	−	−	−	−	−
7	VF smear on jeans, spiked with semen (100 µL 1:100)	−	+	+	−	−	−	+	+
8	4 yr old blood on filter paper	+	+	−	−	−	−	−	−
9	4 yr old semen on filter paper	−	−	+	−	−	+	−	−
10	Nasal swab	−	−	−	−	−	−	−	−
11	Liquid breast milk	−	−	−	−	−	−	−	−
12	Breast milk on swab, spiked with saliva (100 µL)	−	−	−	−	+	+	−	+

## Data Availability

The data presented in this study are available on request from the corresponding author.

## Supplementary Materials

The following supporting information can be downloaded at: https://www.mdpi.com/article/10.3390/mi15020271/s1.
