# Supplementary figures and images for: Three-Dimensional-Printed Instrument for Isothermal Nucleic Acid Amplification with Real-Time Colorimetric Imaging
Source: Micromachines (Basel). 2024 Feb 14;15(2):271. doi: 10.3390/mi15020271 (PMC10892149; doi:10.3390/mi15020271)

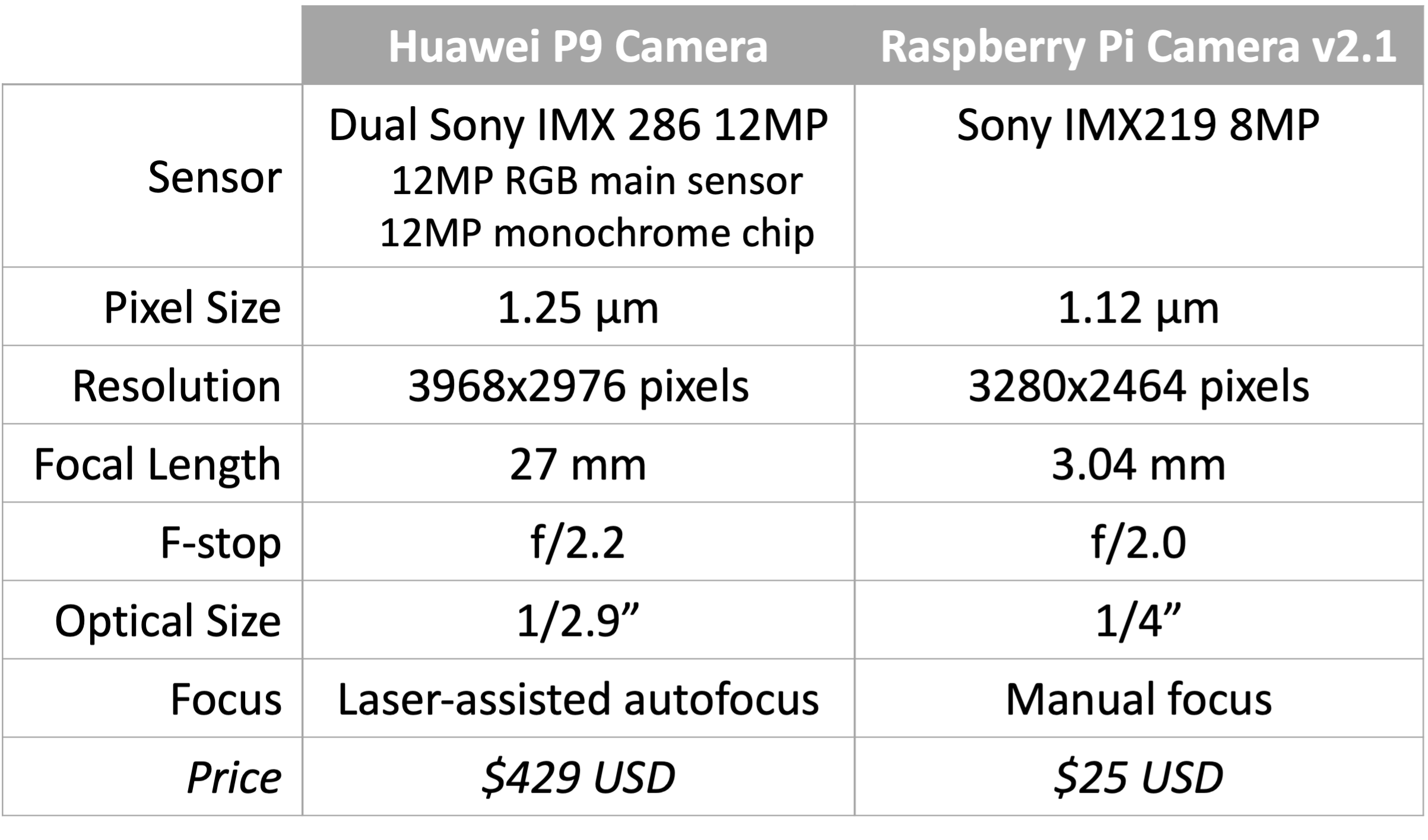

Supplement: Supplementary file 1 [file micromachines-15-00271-s001.zip › Supplementary/Table S1.png]

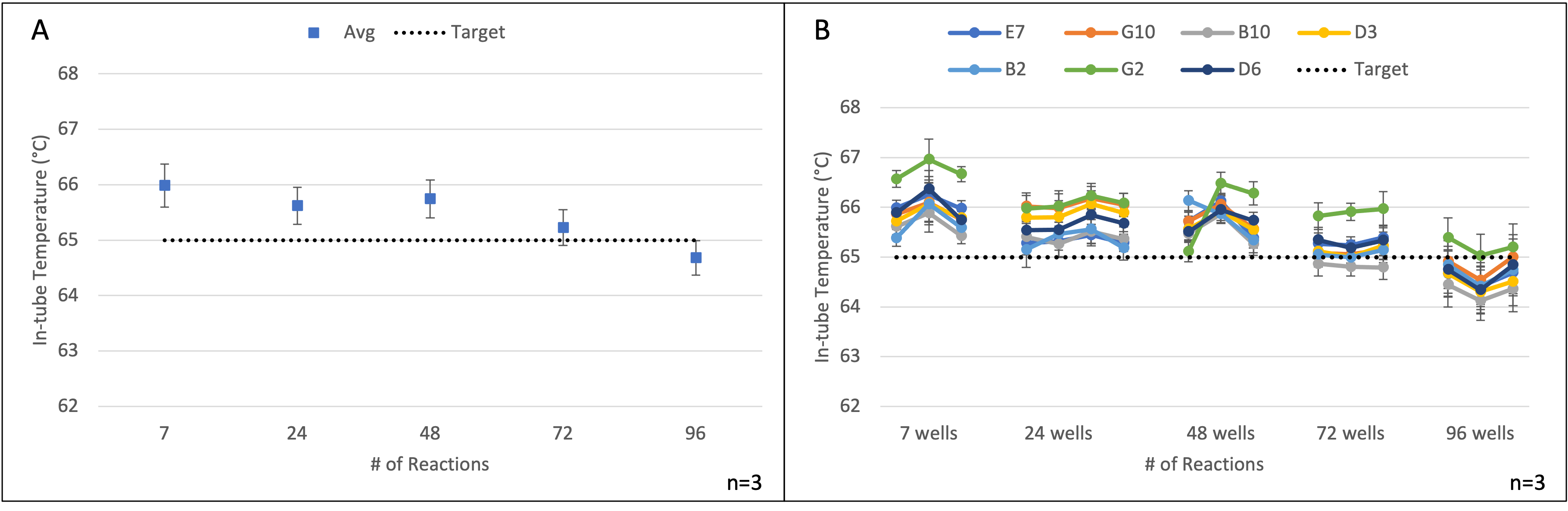

Supplement: Supplementary file 1 [file micromachines-15-00271-s001.zip › Supplementary/Figure S3.png]

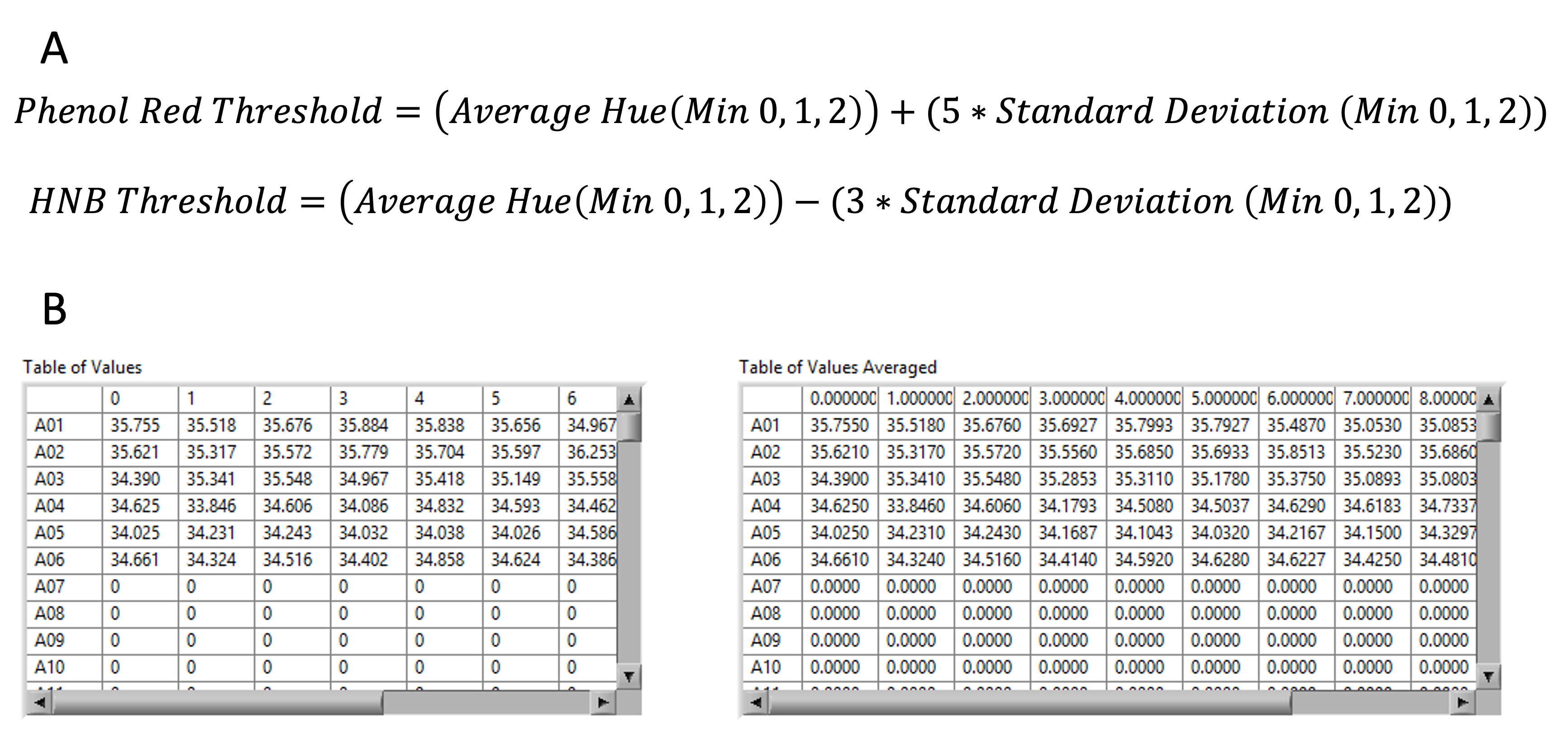

Supplement: Supplementary file 1 [file micromachines-15-00271-s001.zip › Supplementary/Figure S2.png]

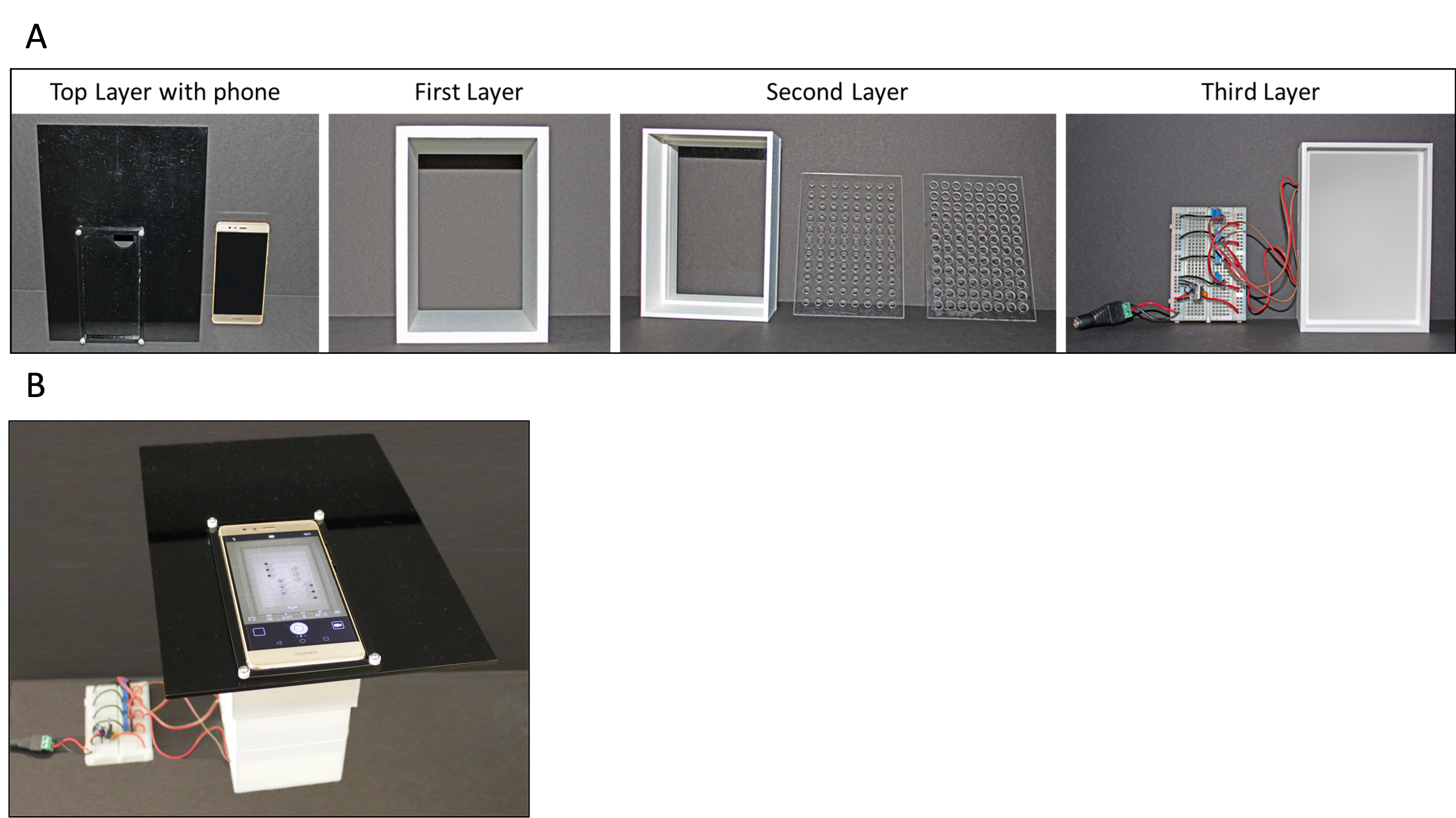

Supplement: Supplementary file 1 [file micromachines-15-00271-s001.zip › Supplementary/Figure S1.png]
